# EFEMP1 as a Potential Biomarker for Diagnosis and Prognosis of Osteosarcoma

**DOI:** 10.1155/2020/5264265

**Published:** 2020-03-19

**Authors:** Zhuo Wang, Jihui Kang, Jiayan Lian, Leilei Huang, Wenlin Xie, Dongliang Zhao, Huisi Ma, Zhongwei Lin

**Affiliations:** ^1^Department of Pathology, The First Affiliated Hospital, Sun Yat-sen University, Guangzhou 510080, China; ^2^Department of Pathology, The Seventh Affiliated Hospital, Sun Yat-sen University, Shenzhen 518000, China; ^3^Department of Cardiology, The First Affiliated Hospital of Guangdong Pharmaceutical University, Guangzhou 510000, China

## Abstract

Osteosarcoma (OS) is the most common primary bone malignancy. Our previous study revealed an association between the level of epidermal growth factor-containing fibulin-like extracellular matrix protein 1 (EFEMP1) and the invasion, metastasis, and poor prognosis of OS. However, the exact correlation between the serum EFEMP1 level and OS diagnosis and progression was unclear. This study is aimed at determining the value of the serum EFEMP1 level in the diagnosis and prognosis of OS. Fifty-one consecutive OS patients were prospectively registered in this study. The serum EFEMP1 levels were measured using ELISA at diagnosis, after neoadjuvant chemotherapy, and before and after surgical treatment. Sixty-nine healthy subjects in the control group, nine patients with chondrosarcoma, and 12 patients with giant cell tumor of the bone were also enrolled in this study. Surgical orthotopic implantation was used to generate a mouse OS model, and the correlation between the circulating EFEMP1 levels and tumor progression was examined. Then, OS patients had significantly higher mean serum EFEMP1 levels (7.61 ng/ml) than the control subjects (1.47 ng/ml). The serum EFEMP1 levels were correlated with the Enneking staging system (*r* = 0.32, *P* = 0.021) and lung metastasis (*r* = 0.50, *P* < 0.001). There was also a correlation between the serum EFEMP1 level and EFEMP1 expression in the respective OS samples (*r* = 0.49, *P* < 0.001). Additionally, patients with either chondrosarcoma or giant cell tumor of the bone had significantly higher serum EFEMP1 levels than OS patients. Surgical and chemotherapeutic treatment led to an increase in the serum EFEMP1 levels. Then, the destruction of bone tissues might be one of the factors about the EFEMP1 levels. In the mouse OS model, the serum EFEMP1 level was correlated with tumor progression. Our results suggested that serum EFEMP1 levels might be used to distinguish OS patients from healthy controls and as an indicator for OS lung metastasis. Serum EFEMP1 levels could serve as a new and assisted biomarker for the auxiliary diagnosis and prognosis of OS.

## 1. Introduction

Osteosarcoma (OS) is the most common primary bone malignancy and is diagnosed through radiological investigations and standard tissue biopsy. Some molecules present in the peripheral blood, such as alkaline phosphatase (AKP) and lactate dehydrogenase (LDH), have been proposed to serve as biomarkers for the diagnosis of OS, but they are not specific for OS and remain on debate with regard to the accuracy and reliability [1, 2]. Recently, serum miRNAs (such as miR-124 [3]) and metabolomics (such as fibrinogen, vascular endothelial growth factor, and basic fibroblast growth factor [4]) were also reported to have diagnostic and prognostic value for OS. Unfortunately, due to the high chromosomal instability and extremely complex karyotypes, none of these candidate biomarkers for OS diagnosis are widely used for clinical purposes. Hence, there is a need to identify a novel reliable biomarker for OS diagnosis and prognosis.

Epidermal growth factor-containing fibulin-like extracellular matrix protein 1 (EFEMP1, also named fibulin-3 or FBLN3), an extracellular matrix glycoprotein, is widely expressed in several developing and adult tissues [5]. Conflicting observations have been reported regarding the expression and role of EFEMP1 in tumors. For example, EFEMP1 expression is upregulated and associated with tumorigenesis in a number of tumors including bladder cancer and glioma [6, 7] and has been reported to promote the growth of human pancreatic adenocarcinoma [8]. On the other hand, downregulation of EFEMP1 was observed in some other malignancies including hepatocellular carcinoma and breast cancers [9, 10]. Our previous study revealed that EFEMP1 was associated with the invasion, metastasis, and poor prognosis of OS [11]. However, the exact correlation between the serum EFEMP1 levels and OS progression remains unclear. This study is aimed at determining the value of the serum EFEMP1 level in the diagnosis and prognosis of OS.

## 2. Materials and Methods

### 2.1. Subject Selection

This prospective study registered a total of 51 OS patients, 69 healthy control subjects, 9 patients with chondrosarcoma, and 12 patients with giant cell tumor of the bone from the First Affiliated Hospital at Sun Yat-sen University between 2014 and 2016. All clinical diagnoses were confirmed by two experienced pathologists through clinical histopathology on all tissue samples obtained from these patients. The control subjects were verified to be healthy based on chest X-rays, liver function tests, and routine physical examinations. All OS patients were treated with neoadjuvant chemotherapy, followed by surgery to remove tumors, and then by postoperative adjuvant chemotherapy. Surgery was the only treatment administered for other primary bone tumor patients. Serum samples were collected from all patients before surgery and were also collected from the 51 OS patients <4 weeks postsurgery and ≥4 weeks postsurgery (the maximum time after surgery was 12 weeks). The order of serum samples from OS patients is pretreatment (presurgery and preneoadjuvant chemotherapy), presurgery and postneoadjuvant chemotherapy, <4 weeks postsurgery, and ≥4 weeks postsurgery. 36-month follow-up survival survey was completed in all OS patients. Tumor tissue specimens (i.e., paraffin-embedded tissue blocks) prepared from patients who underwent surgery were obtained from the Department of Pathology at the First Affiliated Hospital at Sun Yat-sen University based on the registration numbers. We have obtained the statement of patient consent to participate in the study or to use their tissues. The study protocol was approved by the Ethics Committees of the First Affiliated Hospital at Sun Yat-sen University.

### 2.2. ELISA

A sandwich enzyme immunoassay for in vitro quantitative measurement of serum EFEMP1 levels was performed using a standard ELISA kit (SEF422Hu, Cloud-Clone Corp., USA) according to the protocol provided by the manufacturer (https://www.cloud-clone.us/elisa/ELISA-Kit-for-Fibulin-3-(FBLN3)-7067.htm). Briefly, the serum samples were diluted in phosphate-buffered saline (PBS). Afterward, 100 *μ*l of the standard solution or serum sample was added to each well. The measurement was performed based on the manufacturer's protocol. The wavelength of the measurement was 450 nm. Set up 7 points of diluted standard such as 100 ng/ml, 50 ng/ml, 25 ng/ml, 12.5 ng/ml, 6.25 ng/ml, 3.12 ng/ml, and 1.56 ng/ml, and the last EP tubes with standard diluent are the bank as 0 ng/ml (Supplementary information ([Supplementary-material supplementary-material-1]) about standard curve).

### 2.3. Immunohistochemistry

Standard immunohistochemistry procedures were conducted on 5 *μ*m tissue sections prepared from formalin-fixed paraffin-embedded tissue blocks. Briefly, the sections were deparaffinized and rehydrated in xylene and alcohol, followed by antigen retrieval in 0.01 M sodium citrate buffer (pH 6.0) heated in a microwave oven. Afterward, the tissue sections were incubated with an anti-EFEMP1 antibody (1 : 50 dilution, AP9095a, Abgent, San Diego, CA) in a humidified container at 4°C overnight. The negative control sections were incubated with PBS. Thereafter, the tissue sections were incubated with EnVision-HRP secondary antibody (Dako, Carpinteria, CA). At least five randomly selected regions from each tissue section were scored. The integral optical density and area were quantified using Image-Pro Plus 6.0 software. The average of five optical density values was used to represent the expression intensity for each section.

### 2.4. Surgical Orthotopic Implantation for the Establishment of Mouse OS Model

BALB/c nude mice (four to five weeks old; 6 mice per group; purchased from Laboratory Animal Center of Sun Yat-sen University) were used in this study. After the mice were abdominally anesthetized with 4% chloral hydrate (0.4 g chloral hydrate/kg of animal body weight), local anesthesia with 2% lidocaine around the operating organization was carried out, which was the way to assist with anesthesia. A needle (0.3 mm) was inserted into the medullary cavity through the intramedullary canal in the tibia. Afterward, 100 *μ*l of PBS containing 1.5 × 10^5^ 143B cells (a human osteosarcoma cell line (ATCC® CRL­8303™) which come from a 13-year-old female Caucasian) was injected into the intramedullary space of each mouse. The Matrigel was not used for the orthotopic implantation. The size of the tumor and the weight of each mouse were recorded. The mice were euthanized when they appeared extremely emaciated (the body condition score is 1/5 or 2/5 with profoundly lethargic) or the tumor's diameter exceeded 2 cm in mice without the muscle and skin in the study. Blood was drawn from the eyes of the mice abdominally anesthetized with 4% chloral hydrate (0.4 g chloral hydrate/kg of animal body weight) at 10, 20, 30, and 35 days after the mouse OS model was successfully established. Small animal-computerized tomography assisted analysis of the tumor size and bone fracture on the mouse OS models. The serum EFEMP1 levels were determined with an ELISA kit as mentioned above. The mice were sacrificed by cervical dislocation after the blood collection at each time point. After the mice were sacrificed, the tumor tissues were collected and fixed for subsequent analysis. The animal protocols were reviewed and approved by the Guidance of Institutional Animal Care and Use Committee at Sun Yat-sen University.

### 2.5. Statistical Analyses

The serum EFEMP1 levels of the OS patients and control subjects were compared with Wilcoxon rank-sum test. Receiver operating characteristic (ROC) curves were generated to determine the cutoff value for serum EFEMP1 levels and calculate Youden's index, sensitivity, and specificity. Pearson's linear correlation or Spearman's rank correlation was conducted to determine the correlation between the serum EFEMP1 level and other variables. Cox regression models were used to examine the association between the presurgery and preneoadjuvant chemotherapy serum EFEMP1 level and the overall survival of the OS patients. Those variables that were significant at the 0.10 level in univariate analysis or were widely reported in previous studies were simultaneously entered in the Cox regression models. Cox regression models were also used to determine the hazard ratios (HR) and 95% confidence intervals (CI). The Kruskal-Wallis test was used to determine the significant difference in the serum EFEMP1 levels of the OS mice at different time points during OS progression. The Wilcoxon matched pairs signed rank sum test was used to determine the significant difference in the serum EFEMP1 levels of the OS patients at different time points. The Bonferroni correction was used to set the alpha level for paired comparison. All statistical analyses were conducted using SPSS version 22.0 and GraphPad Prism version 5. A two-tailed *P* value of less than 0.05 was considered statistically significant.

## 3. Results

### 3.1. Determination of Serum EFEMP1 as a Biomarker for OS Diagnosis

**Figure 1:**
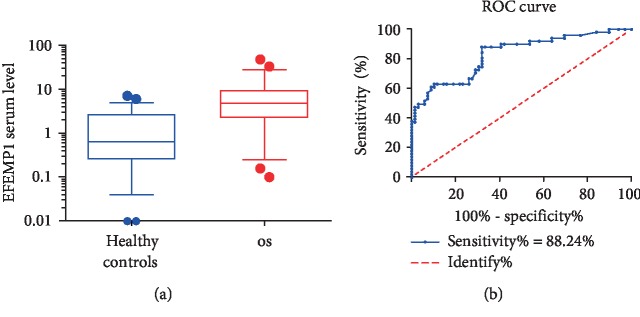
Determination of serum EFEMP1 as a biomarker for OS diagnosis. (a) The serum EFEMP1 level of OS patients was significantly higher than that of the healthy controls (*P* < 0.001). (b) ROC curves identifying serum EFEMP1 as a novel biomarker for OS diagnosis. An optimum EFEMP1 cutoff value is 1.51 when Youden's index equals 56.36% (sensitivity = 88.24%, specificity = 68.12%).

A total of 51 OS patients and 69 healthy controls were enrolled in this study. The mean serum EFEMP1 level in the OS group (standard deviation, SD) was 7.61 (8.76) ng/ml, which was significantly higher than that of the healthy controls (1.47 (1.65) ng/ml) (Figure 1(a), *P* < 0.001, Supplementary [Supplementary-material supplementary-material-1]). We next used ROC curves to assess the potential use of serum EFEMP1 as a noninvasive biomarker for OS diagnosis. As shown in Figure 1(b), based on Youden's J statistic (Youden′s index = sensitivity + specificity − 100%) for the diagnosis of OS patients, we determined an optimum EFEMP1 cutoff value, which is 1.51 when Youden's index equals 56.36% (Supplementary [Supplementary-material supplementary-material-1]). The area under the curve (AUC) for OS was 0.83 (95%CI = 0.76 to 0.91, *P* < 0.001), with a sensitivity of 88.24% and a specificity of 68.12% (Figure 1(b)).

We also used Spearman's rank correlation analysis to examine the correlation between the serum EFEMP1 level and the clinicopathological parameters (Table 1). Among these 51 patients, there was a correlation between the Enneking staging system and the serum EFEMP1 level (*r* = 0.32, *P* = 0.021). There was also a significant correlation between the Enneking staging system and tumor size (*r* = 0.59, *P* < 0.001). The correlation between gender, age, tumor position, tumor size, histologic type, and serum EFEMP1 levels in OS patients is not statistically significant (*P* > 0.05).

### 3.2. Comparison of Serum EFEMP1 between OS Patients and Other Primary Bone Tumor Patients

To study the specificity of serum EFEMP1 for OS, we compared the serum EFEMP1 level between OS patients and patients with other primary malignant bone tumors, chondrosarcoma, and giant cell tumor of the bone. The mean serum level of EFEMP1 in chondrosarcoma patients and patients with giant cell tumor of the bone (SD) was 12.53 (7.56) ng/ml (Supplementary [Supplementary-material supplementary-material-1]), which was significantly higher than that of the healthy controls (*P* < 0.001). Additionally, patients with either chondrosarcoma or giant cell tumor of the bone had significantly higher serum EFEMP1 levels than OS patients (*P* = 0.002). Thus, an increased circulating EFEMP1 level is not specific for OS in many primary bone tumors.

### 3.3. Correlation between Serum EFEMP1 Level and EFEMP1 Expression in Tumor Tissues of OS Patients

**Figure 2:**
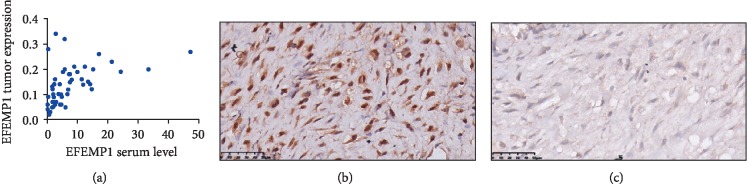
Correlation between serum EFEMP1 level and EFEMP1 expression in tumor tissues of OS patients. (a) Scatter plot of correlation analysis between the serum EFEMP1 level (ng/ml) and EFEMP1 expression in tumor tissues. (b) Representative immunohistochemical images showing strong EFEMP1 staining in tumor tissues. (c) Representative immunohistochemical images showing weak EFEMP1 staining in tumor tissues.

We next used Pearson's correlation analysis to examine the correlation between the serum EFEMP1 level and the EFEMP1 expression in the respective tumor samples evaluated using immunohistochemical staining and found that there was a strong correlation between the two (*r* = 0.49, *P* < 0.001, Figure 2).

### 3.4. Comparison of Serum EFEMP1 Level between Pre- and Postsurgical OS Patients

**Figure 3:**
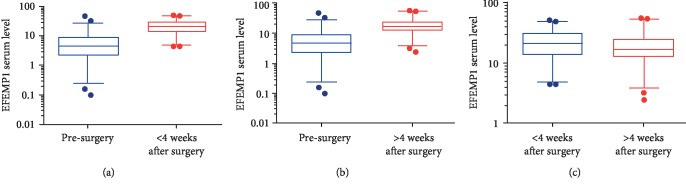
Comparison of serum EFEMP1 level between pre- and postsurgical OS patients. (a) The serum EFEMP1 level of OS patients < 4 weeks after surgery was significantly higher than that before surgery (*P* < 0.001). (b) The serum EFEMP1 level of OS patients > 4 weeks after surgery was significantly higher than that before surgery (*P* < 0.001). (c) The serum EFEMP1 level of OS patients < 4 weeks after surgery was significantly higher than that >4 weeks after surgery (*P* = 0.012).

Surgery is the mainstay of treatment for OS patients. To examine the effect of surgery on the serum EFEMP1 levels of OS patients, we first measured and compared the circulating levels of EFEMP1 in OS patients pre- and post- (<4 weeks after) surgery. We found that the serum EFEMP1 level in postsurgical OS patients (i.e., within four weeks postsurgery) was 22.83 (11.92) ng/ml (mean (SD)), which was significantly higher than that of OS patients before surgery (7.61 (8.76) ng/ml (mean (SD)), *P* < 0.001, Figure 3(a), Supplementary [Supplementary-material supplementary-material-1]). Similarly, the serum EFEMP1 level in OS patients at ≥4 weeks postsurgery was 19.93 (12.10) ng/ml (mean (SD)), which was significantly higher than that before surgery (*P* < 0.001, Figure 3(b), Supplementary [Supplementary-material supplementary-material-1]). We also compared the serum EFEMP1 level in OS patients within four weeks postsurgery with the level ≥ 4 weeks postsurgery and found that the former was significantly higher (*P* = 0.012, Figure 3(c)). Taken together, surgery increased the serum EFEMP1 levels in OS patients, and OS patients during the early phase postsurgery (i.e., within four weeks after surgery) had higher circulating EFEMP1 levels than OS patients in the later phase postsurgery (i.e., ≥4 weeks postsurgery).

### 3.5. Comparison of Serum EFEMP1 Levels between Pre- and Postneoadjuvant Chemotherapy OS Patients

Our OS patients underwent neoadjuvant chemotherapy. Given the above findings that surgery increased serum EFEMP1 level in OS patients, we compared the circulating EFEMP1 level in OS patients before and after neoadjuvant chemotherapy. We found that the serum EFEMP1 level of OS patients before neoadjuvant chemotherapy was 7.61 (8.76) ng/ml (mean (SD)), which was significantly lower than that after neoadjuvant chemotherapy (20.18 (13.68) ng/ml and presurgery (mean (SD)), *P* < 0.001, Supplementary [Supplementary-material supplementary-material-1]). Hence, neoadjuvant chemotherapy also elevates the serum EFEMP1 levels of OS patients, similar to surgery.

### 3.6. Association between Serum EFEMP1 Level and Lung Metastasis of OS

The lung metastasis of OS was verified based on chest X-rays and computerized tomography. We used Spearman's rank correlation analysis to examine the correlation between the serum EFEMP1 level and the lung metastasis of OS at the time of blood sampling and found a significant correlation between these two (*r* = 0.50, *P* < 0.001). The “follow-up with lung metastasis” was also taken into account. “Follow-up with lung metastasis” meant the appearance of new metastatic lesions on the lung during the postsurgery phase (36-month follow-up survival survey). There was also a significant positive correlation between the serum EFEMP1 level and follow-up with lung metastasis (*r* = 0.35, *P* = 0.012). Hence, the serum EFEMP1 level is positively correlated with lung metastasis of OS.

### 3.7. Association between Serum EFEMP1 Level and Overall Survival of OS Patients

We next examined the association between the serum EFEMP1 level and the overall survival of OS patients. The Cox regression equation was employed to identify the potential factors that affected the overall survival of OS patients. After the correction of some covariates (sex, age, tumor position, tumor size, metastasis, and histologic type) by Cox regression, no significant correlation between the serum EFEMP1 level and the overall survival of OS patients was detected (corrected HR = 0.60, 95%CI = 0.22‐1.65). However, there was a significant correlation between follow-up with lung metastasis and the overall survival of OS patients (corrected HR = 3.60, 95%CI = 1.01‐2.99) (Table 2).

### 3.8. Association between Serum EFEMP1 and Tumor Progression in OS Mice

**Figure 4:**
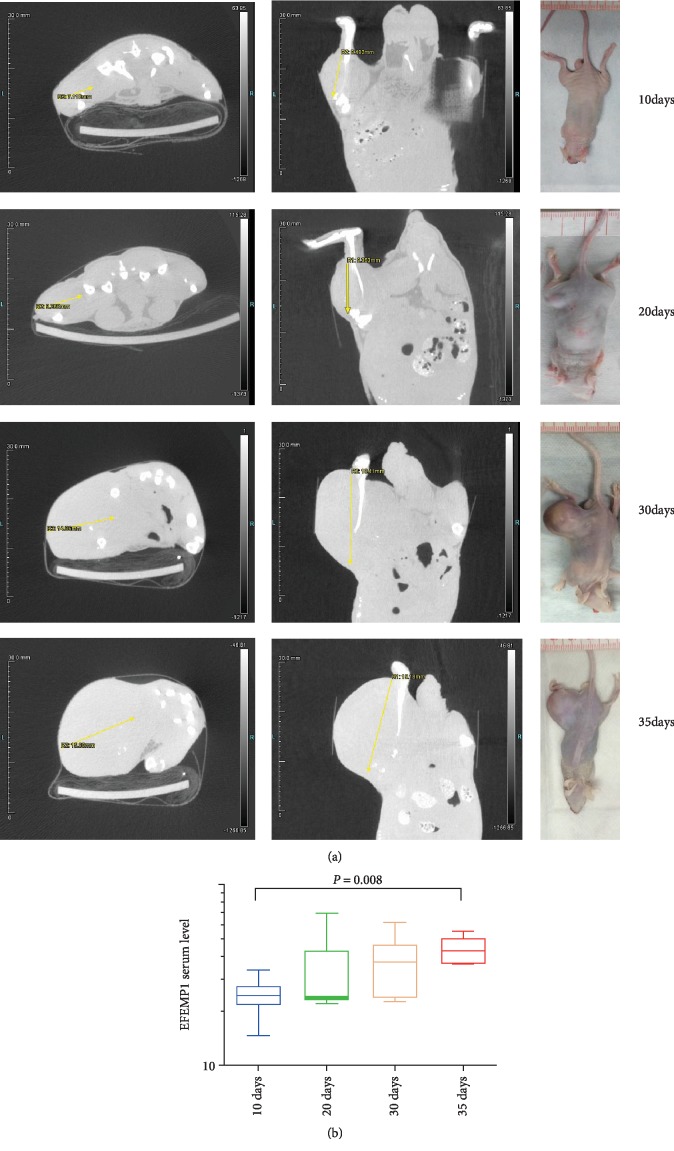
Association between serum EFEMP1 and tumor progression in OS mice. (a) Images of small animal-computerized tomography showing the 10-, 20-, 30-, and 35-day tumor groups of OS mice. (b) The serum EFEMP1 level of the 35-day group was significantly higher than that of the 10-day group (*Z* = −2.657, *P* = 0.008).

Tumor groups (10, 20, 30, and 35 days) for OS mice were established corresponding to 10-, 20-, 30-, and 35-day OS patients. Serum samples were collected at different time points (10, 20, 30, and 35 days, respectively) after tumor development. We found that the tumor size gradually increased with the progression of the tumor as expected, which coincided with a gradual increase in the serum EFEMP1 levels (Figure 4). The serum EFEMP1 level from the 10-day, 20-day, 30-day, and 35-day tumor group of mice was 24.40 (6.40), 23.80 (20.95), 37.40 (23.50), and (14.98) ng/ml (median (quartile spacing)). There was a statistically significant difference in the serum EFEMP1 levels between the four time points evaluated (*H* = 8.345, DF = 3, *P* = 0.039). The serum EFEMP1 level of the 35-day group was significantly higher than that of the 10-day group (*Z* = −2.657, *P* = 0.008) (Figure 4), but there was no significant difference between the other two groups. Collectively, an increase in the serum EFEMP1 level was associated with the progression of OS in the animal model.

## 4. Discussion

The present study examined the potential value of serum EFEMP1 as a biomarker for the diagnosis of OS and the prediction of OS metastasis in both human patients and the animal OS model. The major findings included that (1) OS patients had significantly higher serum EFEMP1 levels than the healthy controls, (2) an increase in the serum EFEMP1 levels was associated with lung metastasis in OS patients, (3) an increase in the serum EFEMP1 levels was associated with tumor progression in the animal model, and (4) there was a correlation between the serum EFEMP1 level and Enneking staging of OS.

EFEMP1 has been shown to be upregulated in pleural mesothelioma and is regarded as a potential biomarker for the disease [12, 13]. Additionally, the EFEMP1 levels in the cerebrospinal fluid and serum samples of meningioma patients are significantly higher compared to those of controls [14], and a high serum EFEMP1 level is associated with poor prognosis for ovarian cancer [15]. However, the EFEMP1 levels in the serum and urine of patients with prostate and colon cancer are significantly lower compared with those of control subjects [16, 17]. Hence, EFEMP1 may either promote or suppress tumor growth, depending on the context. Previous research indicated that EFEMP1 expression was profoundly reduced in articular cartilage with ageing [18] and that two EFEMP1 subtypes, fibulin3-1 and fibulin3-2, were potential biomarkers for the diagnosis of osteoarthritis [19]. These findings suggest that serum EFEMP1 levels may be altered in patients with diseases involving the bone. Indeed, in the present study, OS patients had significantly higher circulating levels of EFEMP1 compared with healthy controls, consistent with our previous report [11]. We also found that the serum EFEMP1 levels in patients with chondrosarcoma were higher compared to those of controls, suggesting that EFEMP1 was potentially involved in the process of chondrogenic differentiation. However, the correlation between EFEMP1 and chondrosarcoma remains unclear and warrants further investigation in the future.

We found that there was a positive correlation between the serum EFEMP1 level and OS staging. Moreover, in the animal model, the progression of OS was accompanied by a gradual increase in the serum EFEMP1 level. Given that OS patients had significantly higher EFEMP1 levels than healthy subjects, we speculated that increased EFEMP1 levels were associated with the progression of OS. However, we did not find a significant link between the serum EFEMP1 level and the overall survival of OS patients, though significant positive associations between the serum EFEMP1 level and lung metastasis as well as between lung metastasis and overall survival in OS patients were detected. Thus, the negative finding regarding the correlation between the serum EFEMP1 level and the overall survival of OS patients was probably due to the limited sample size in this study. Mechanistically, how EFEMP1 is released into the peripheral blood remains unclear. EFEMP1 has been reported in the study of exosome of nasopharyngeal carcinoma [20]. Then, it is likely that EFEMP1 is secreted either from the extracellular exosomes or from dying tumor tissues. In our previous study, EFEMP1 was specifically upregulated in OS [11]. Here, we found that there was a strong correlation between the serum EFEMP1 level and the characteristics of the OS samples. Further, serum EFEMP1 increased gradually with the progression of OS in the OS animal model. Hence, one potential mechanism is the secretion of serum EFEMP1 from OS tumor cells.

As mentioned above, OS patients had significantly higher circulating levels of EFEMP1 than healthy control subjects. Further analysis revealed an AUC of 0.83 for predicting the presence of OS, which is comparable to those of the current clinically used OS biomarkers including AKP, LDH, and miR-124 [2, 3]. Although the serum EFEMP1 levels in OS patients and healthy controls were significantly different, EFEMP1 is not a specific biomarker for OS that can be used to distinguish OS from other skeletal system tumors such as chondrosarcoma and giant cell tumor of the bone. The serum EFEMP1 level was affected by bone damage. However, we believe that the serum EFEMP1 level may be used to assist in the diagnosis of OS to distinguish the normal population, but the gold standard for definitive diagnosis of OS should still be obtained through histopathology. A higher serum EFEMP1 level would indicate more serious bone destruction. As the destruction of bone tissues by OS increases, the serum EFEMP1 level would increase, in addition to tissue damage by treatments such as surgery and neoadjuvant chemotherapy.

Some limitations of this study should be noted. For example, this was a single-center clinical study with a small sample size. It caused the coefficient of variation presented in our result to be a little high in the groups. If the sample size could be increased, the coefficient of variation might be reduced. Moreover, it might be helpful to reduce frozen storage time of samples and avoid repeated freeze/thaw cycles. Additionally, while the correlation between the serum EFEMP1 level and the progression of OS was established in the animal model, whether it holds true in OS patients remains uncertain for the time being. In addition, we did not simultaneously examine and compare the correlation between other OS biomarkers and serum EFEMP1 in this study. In observational studies, follow-up with lung metastasis often occurs sometime after initiation of a study. Previous research has shown that immortal-time bias may appear in the medical literature and frequently affect key factors. Thus, large cohort studies in the future should be carried out to clarify our findings.

## 5. Conclusions

We demonstrated in the present report that the serum EFEMP1 level may be used to distinguish OS patients from healthy controls and as an indicator for the possibility of OS lung metastasis. An increase in the serum EFEMP1 level of OS patients may be associated with OS progression. Our findings suggest that the serum EFEMP1 level may serve as a novel and assisted biomarker for the auxiliary diagnosis of OS and the prediction of OS metastasis.

## Figures and Tables

**Table 1:** Correlation between serum EFEMP1 level and clinicopathological parameters of OS^a^.

Variable	EFEMP1 serum level	Gender	Age	Tumor position	Tumor size	Enneking staging	Histologic type
EFEMP1 serum level	1.00	—	—	—	—	—	
Gender	0.08	1.00	—	—	—	—	
Age	0.15	-0.16	1.00	—	—	—	
Tumor position	0.06	-0.06	0.23	1.00	—	—	
Tumor size	0.22	-0.10	0.02	-0.06	1.00	—	
Enneking staging	**0.32** ^∗^	-0.24	0.15	0.06	**0.59** ^∗∗∗^	1.00	
Histologic type	**0.03**	0.07	0.01	-0.16	-0.08	-0.16	1.00

^a^Spearman's rank correlation analysis; ^∗^*P* < 0.05; ^∗∗∗^*P* < 0.001.

**Table 2:** Correlation between serum EFEMP1 level and overall survival of OS patients.

Variable	Overall survival state (survival = 0, dead = 1)	
	HR^a^ (95% CI)	*P* value
EFEMP1 serum level^#^		
<4.69 ng/ml		
≥4.69 ng/ml	0.62 (0.22-1.77)	0.371
Gender		
Female	1.00	
Male	0.71 (0.27-1.85)	0.486
Age		
<20 years	1.00	
≥20 years	0.99 (0.37-2.69)	0.984
Tumor position		
Bones of the limbs	1.00	
Bones outside the limbs	1.08 (0.40-2.94)	0.884
Tumor size		
≤8 cm	1.00	
>8 cm	0.69 (0.22-2.12)	0.516
Follow-up with lung metastasis		
No	1.00	
Yes	3.46 (0.89-13.42)	0.073
Lung metastasis at the time of blood sampling		
No	1.00	
Yes	1.90 (0.54-6.67)	0.317
Histologic type		
Conventional OS (osteoblastic)	1.00	
Conventional OS (chondroblastic)	0.81 (0.24-2.72)	0.738
Conventional OS (fibroblastic)	0.68 (0.18-2.62)	0.580
Not conventional OS (telangiectatic or small cell OS)	0.73 (0.09-6.12)	0.770

HR: hazard ratios; CI: confidence intervals. ^a^Removal of tumor staging HR. ^#^Group by median.

## Data Availability

The data used to support the findings of this study are available from the corresponding author upon request.

## Supplementary Materials

Supplementary Materials There are three materials in the supplementary materials, including a standard curve for one test in the study, the summary table of serum EFEMP1 level for all the group, and the contingency table of predicted and true OS diagnosis using EFEMP1 cutoff 1.51.
