# Supplementary material for: EFEMP1 as a Potential Biomarker for Diagnosis and Prognosis of Osteosarcoma
Source: Biomed Res Int. 2020 Mar 19;2020:5264265. doi: 10.1155/2020/5264265 (PMC7115049; doi:10.1155/2020/5264265)
Supplement: Supplementary Materials — There are three materials in the supplementary materials, including a standard curve for one test in the study, the summary table of serum EFEMP1 level for all the group, and the contingency table of predicted and true OS diagnosis using EFEMP1 cutoff 1.51. [file 5264265.f1.pdf]

Supplementary information Standard curve for one test in the study

	Conc（ng/ml）	OD mean	OD1	OD2
S1	0	0.053	0.048	0.058
S2	1.56	0.0585	0.057	0.060
S3	3.12	0.0705	0.071	0.070
S4	6.25	0.0885	0.088	0.089
S5	12.5	0.1625	0.160	0.165
S6	25	0.3705	0.361	0.380
S7	50	0.682	0.677	0.687
S8	100	1.466	1.501	1.431

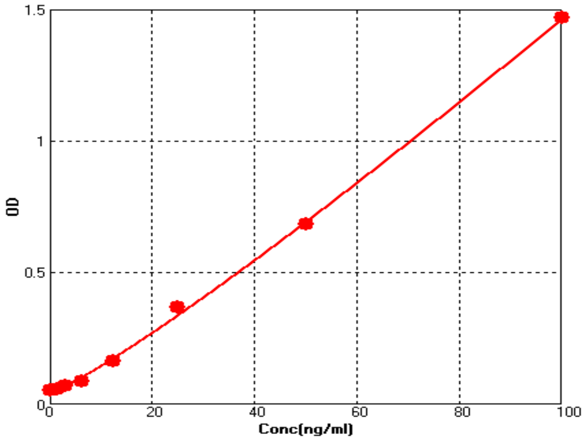

Standard curve

4 Parameter Logistic

==

y = (A - D) / [1 + (x/C)^B] + D

A = 85.01636

B = -1.13959

C = 3568.88171

D = 0.04320

r^2 = 0.99901

==

Supplementary Table 1 Serum EFEMP1 level for all the group of samples analyzed by the ELISA assay

Group	Median (ng/ml)	Quartile spacing (ng/ml)	Mean (ng/ml)	Standard deviation (SD)	Coefficient of variation (CV)
OS group(pre-treatment)	4.69	7.72	7.61	8.76	1.15
healthy controls	0.64	2.52	1.47	1.65	1.12
other primary bone tumor patients (including chondrosarcoma and giant cell tumor of bone)	12.94	11.01	12.53	7.56	0.60
OS pre- surgery and post-neoadjuvant chemotherapy	15.52	18.12	20.18	13.68	0.68
OS patients at < 4 weeks post-surgery	21.17	18.48	22.83	11.92	0.52
OS patients at ≥ 4 weeks post-surgery	16.80	12.85	19.93	12.10	0.61

Supplementary Table 2 Contingency table

Contingency table of predicted and true OS diagnosis using EFEMP1 cutoff 1.51.

		Actual	
		Positive	Negative
Predicted	Positive	45	22
	Negative	6	47
